# Differences in meat and wool quality and gastrointestinal/hypothalamic functions in Dexin fine-wool meat sheep with divergent residual feed intake

**DOI:** 10.1186/s12917-026-05562-0

**Published:** 2026-06-04

**Authors:** Ziting Wang, Xu Wang, Qiyuan Deng, Yong Tuo, Yan Ma, Linjiao He, Zhijun Zhang, Wenxin Zheng

**Affiliations:** 1 Feed Research Institute of Xinjiang Academy of Animal Husbandry Sciences, Urumqi, 830011 China; 2 College of Juncao and Ecology, Fujian Agriculture and Forestry University, No. 15, Shangxiadian Road, Cangshan District, Fuzhou, 350002 Fujian Province China; 3 Xinjiang Academy of Animal Husbandry Sciences, Urumqi, 830011 China; 4 Xinjiang Feed Biotechnology Key Laboratory, Urumqi, 830011 China

**Keywords:** RFI, Hypothalamus-Gastrointestinal, Lnc-RNA, mRNA, Livestock quality

## Abstract

Residual feed intake (RFI) is an important indicator for measuring feed efficiency and can be used to screen sheep populations with low feed consumption and excellent growth performance. Its mechanism is closely related to the hypothalamus, which regulates animal feeding behavior, as well as the rumen and duodenum, which are responsible for the main digestive and absorptive functions. In theory, the hypothalamus-gastrointestinal axis is one of the key pathways affecting feed efficiency in ruminants. However, existing research has focused on the gastrointestinal microbiota and lacks systematic research on the interaction mechanism and functional expression differences between the hypothalamus and the gastrointestinal axis. The results indicate that Residual Feed Intake (RFI) significantly influences livestock quality in sheep. Regarding meat characteristics, the low-RFI (L-RFI) group exhibited a significantly higher cooking percentage in the longissimus dorsi muscle compared to the high-RFI (H-RFI) group. In terms of wool quality, L-RFI sheep were characterized by finer fiber diameters, shorter staple lengths, and an increased straight fiber rate. Mechanistically, RFI affected glutamatergic synapses, calcium signaling, and neurotransmitter receptor activity in the hypothalamus. Transcriptomic profiling further revealed that high-intake sheep showed upregulated lysosomal function, ATP metabolism, and cell cycle pathways in the rumen. Similarly, the duodenum of H-RFI sheep displayed marked activation of DNA replication and B-cell receptor signaling, alongside enhanced cholesterol transport and epithelial cell proliferation. In summary, this study, through a multi-dimensional analysis from the hypothalamus to the gastrointestinal tract, systematically reveals the physiological and molecular basis of RFI’s influence on sheep feed efficiency, providing a new research paradigm for further exploring the role of the brain-gut axis in the regulation of animal feed efficiency.

**Supplementary Information:**

The online version contains supplementary material available at 10.1186/s12917-026-05562-0.

## Introduction

With the improvement of socio-economic conditions and the transformation of consumption patterns, consumers’ requirements for the quality of mutton and wool products have steadily increased. In the large-scale and intensive sheep farming system, feed costs account for 65% to 70% of the total production cost, becoming a key factor affecting economic benefits [1]. Therefore, improving feed conversion efficiency has become an important goal in modern sheep breeding and farming practices. For a long time, feed conversion ratio (FCR) has been widely used as a core indicator for evaluating livestock feed utilization efficiency. FCR is easy to calculate, the results are intuitive, and it facilitates the summarization and comparison of production data.However, with the expansion of ruminant farming, the limitations of FCR have become increasingly apparent: it cannot distinguish between individuals with different production performance, such as “high feed intake-high weight gain” and “low feed intake-low weight gain,” resulting in significant biases in economic benefit assessment. To more accurately reflect individual differences, Residual Feed Intake (RFI), as a more scientific negative evaluation indicator, has gradually gained industry attention. Compared to FCR, RFI is not only negatively correlated with feed efficiency but can also effectively assess individuals with negative daily weight gain, thus possessing better applicability in evaluating ruminant production performance.

Currently, research on RFI and the physiological mechanisms of ruminants mainly focuses on the microbial community. Studies have shown that rumen fermentation patterns and microbial composition can explain about 19%–20% of RFI variation [2, 3]. Ellison et al. [4] found that six microorganisms in the rumen of ewes were highly correlated with actual feed efficiency, suggesting that specific microorganisms may play a key role in feed efficiency regulation.In the duodenum, differentially expressed genes related to RFI mainly involve lipid metabolism, protein synthesis and immune regulation. Other experiments have shown that SLC9A1, HIF1A and ACO2 are expressed more in rumen epithelial cells of low-RFI bovine cells [5]. Transcriptome analysis of the liver and ileum of fattening lambs further revealed that feed efficiency is closely related to gluconeogenesis, tricarboxylic acid cycle, protein synthesis, cholesterol metabolism, and inflammatory processes.This shows that energy metabolism, protein synthesis, and immune function together determine the feed conversion efficiency of ruminants.However, current research on how the hypothalamus regulates the function of liver, duodenum and rumen epithelial cells through the brain-gut axis is still relatively scarce, mainly due to factors such as the long RFI test cycle and high detection cost.Therefore, systematically exploring the differences in the molecular regulatory mechanisms of the hypothalamus-gastrointestinal tract in sheep at different RFI levels and evaluating their impact on meat and wool quality has important theoretical value and practical significance for the efficient development of the sheep industry.

## Materials and methods

All animal procedures were approved by the Agreement Management and Review Committee of the Feed Research Institute of the Xinjiang Academy of Animal Sciences (Approval No. 2 20230620).

### Experimental animals and experimental period

The experiment was conducted at the Baicheng County Breeding Sheep Farm in Xinjiang. A total of fifty 70-day-old Dexin fine-wool meat sheep were obtained from the same farm. Prior to the formal trial, all lambs were dewormed twice and weighed (initial body weight: 30.77 ± 3.17 kg). The experimental period lasted 100 days, including a 14-day adaptation period. During the trial, the sheep were weighed every 20 days, and all animals were slaughtered on the 100th day for subsequent analyses.

### Diet formulation and processing

The feed used was 678S-2 pellet feed provided by Tiankang Feed Technology Co., Ltd. The feed formula and nutritional level of the pellet feed are shown in Table 1. The dry matter (DM) of feed is determined by “GB/T 6435 − 2014 Determination of moisture in feed”, the crude protein (CP) is determined by “GB/T 6432 Determination of crude protein in feed - Kjeldahl method”, the crude fat (EE) is determined by “GB/T 6433 Determination of crude fat in feed”, the crude ash (Ash) is determined by “GB/T 6438 Determination of crude ash in feed”, the calcium (Ca) is determined by “GB/T 13885 Determination of calcium, copper, iron, magnesium, manganese, potassium, sodium and zinc content in feed - atomic absorption spectrometry”, the phosphorus (P) is determined by “GB/T 6437 Determination of total phosphorus in feed - spectrophotometry”, the neutral detergent fiber (NDF) is determined by “GB/T 20806 Determination of neutral detergent fiber (NDF) in feed”, and the acid detergent fiber (ADF) is determined according to “NY/T 1459 Determination of acid detergent fiber in feed.

**Table 1:** Feed nutritional composition

Items		Content
Ingredients		
Corn stover.	13.40	
Alfalfa Powder.	14.35	
Soybean meal.	11.48	
Cottonseed meal.	12.44	
Corn	38.00	
Flour	6.70	
Rock flour	1.0	
Calcium bicarbonate	0.19	
Sodium bicarbonate	0.77	
Sodium chloride	1.0	
Premix	0.67	
Nutrient levels^2^		
Dry Matter (DM)	87.10	
Crude Protein (CP)	16.17	
Crude Fat (EE)	1.83	
Crude Ash (Ash)	11.65	
Neutral Detergent Fiber (NDF)	36.89	
Acid Detergent Fiber (ADF)	29.85	
Calcium (Ca)	0.69	
Phosphorus (P)	0.39	
Metabolizable Energy (ME) MJ/ kg	15.71	

Each kg of premix contains; VA 150 000 IU, VD3 56 500 IU, VE 8 000 IU, Se (sodium selenite) 14 mg, I (potassium iodide) 80 mg, Cu (potassium sulfate) 290 mg, Mn (manganese sulfate) 1 925 mg, Zn (zinc oxide) 2 050 mg, Co (cobalt sulfate) 24 mg. Each kilogram of premix contains: Per kg of the diet: VA 150 000 IU, VD3 56 500 IU, VE 8 000 IU, Se (as sodium selenite) 14 mg, I (as Potassium iodide) 80 mg, Cu (as coPPer sulfate) 290 mg, Mn (as manganese sulfate) 1 925 mg, Zn (as zinc) oxide) 2 050 mg, Co (as cobalt sulfate) 24 mg

Metabolizable energy is a calculated value. The ME of mutton sheep was calculated according to the Mutton Sheep Feeding Standard [6] (NY/T816-2021): ME of mutton sheep (MJ/kg DM) = DE (MJ/kg DM) × 0.82 + 0.046, DE (MJ/kg DM) = 17.211 − 0.135 × NDF (% DM)

### Trial management

Prior to the experiment, all pens were thoroughly disinfected, and the sheep were individually marked with ear tags. Before the pre-feeding period, the animals were dewormed via intramuscular injection and administration of anthelmintics in the feed. Sheep were housed individually and fed twice daily at 10:00 and 18:00, with ad libitum access to water. Feed intake was monitored to ensure that daily leftovers for each sheep exceeded 15% of the provided feed. During the experiment, three sheep were removed from the study due to illness.

### Measurement indicators and methods

#### Calculation and grouping of residual feed intake

SPSS26.0 analysis showed that the R2 values of dry matter intake, daily weight gain and average mid-term metabolic body weight of the experimental sheep met the conditions of the regression equation. A linear model was constructed to calculate the RFI of the experimental sheep. The model is as follows:

$$Yi=\beta 0+\beta 1(ADGi)+\beta 2MBWi+ei$$

where the average daily weight gain formula ADGi=(FBWi-IBWi)/N is calculated, FBWi (Final Boby Weight) is the final body weight of individual i, IBWi (Initial Body Weight) is the initial body weight of individual i, and N is the number of experimental days; and the average metabolic body weight formula is MBWi=【1/2×(FBWi་IBWi)】0.75; β0 represents the regression intercept, β1 and β2 are fixed values, and ei is the RFI of sheep i [1]. According to the mean value and standard deviation of RFI, the test sheep were divided into H-RFI group (RFI> Mean + 0.5SD) with 11 sheep, M-RFI group (Mean + 0.5SD< RFI< Mean-0.5SD) with 18 sheep and L-RFI group (RFI< Mean-0.5SD) with 13 sheep.

#### Data collection

Animals were slaughtered at the slaughterhouse before feeding on the 100th day of the experimental period. Sampling was performed by incision of the neck. The longissimus dorsi muscle was immediately collected to determine meat color and pH on site. Samples of the longissimus dorsi were then bagged, rapidly frozen, and stored at − 20 °C for subsequent laboratory analyses of meat quality, including cooked meat rate, drip loss, water loss rate, and tenderness. Wool from the neck and shoulder was collected, sealed in bags, stored at − 20 °C, and sent for quality testing.

The eye muscle area was measured at the cross-section of the longissimus dorsi between the 12th and 13th ribs. The surface was traced onto sulfuric acid paper and outlined with a pencil. Eye muscle area (cm²) was calculated as: eye muscle width (cm) × eye muscle height (cm) × 0.7.

PH measurements were taken 45 min post-slaughter using approximately 10 g of longissimus dorsi tissue and a pH meter. Meat color was determined under normal daylight conditions using a TC-PⅡG fully automatic colorimeter according to the manufacturer’s instructions [7].

For cooked meat rate, approximately 100 g of psoas muscle was trimmed of external membranes and fat and weighed (W1). The sample was steamed for 50 min, rested for 30 min, and weighed again (W2). Cooked meat rate (%) was calculated as W2/W1 × 100% [8]. Drip loss was assessed using a 5 × 3 × 2 cm longissimus dorsi sample along the muscle fiber direction, stored at 2–4 °C for 24 h in a drip loss funnel. The sample was blotted dry and weighed (W2), with drip loss (%) calculated as (W1 − W2)/W1 × 100% [9]. Water loss rate was determined by compressing a 5 cm-diameter meat sample under 35 kg pressure for 5 min, then weighing before (W1) and after compression (W2); water loss rate (%) = (W1 − W2)/W1 × 100% [6].

Fatty acid composition of the left longissimus dorsi was analyzed by Hepu Lianke Biotechnology Co., Ltd., following national standard GB 5009.168–2016 [10] for long-chain and short-chain volatile fatty acids. Amino acid profiles were determined by Nomi Metabolic Biotechnology Co., Ltd. using LC-MS/MS [11].

Wool samples were analyzed at the Breeding Sheep and Wool Cashmere Quality Supervision and Testing Center of the Ministry of Agriculture and Rural Affairs (Urumqi). Wool fiber diameter, hair length, curl number, straightness, grease content, and color (L, Wht, YI) were measured according to GB/T 21,030 − 2007 and GB/T 1523–2013.

Tissue samples (1 cm × 1 cm) from the hypothalamus, rumen, and duodenum of sheep in the L-RFI and H-RFI groups were collected, placed in 2 mL cryovials, rapidly frozen in liquid nitrogen, transported to the laboratory, and sent to Biocloud Biotech (www.biocloud.net) for transcriptome analysis.

### Data analysis

#### RNA quantification and qualitative analysis

Monitor RNA degradation and contamination, especially DNA contamination, on 1.5% agarose gels. Measure RNA concentration and purity using advanced molecular biology equipment to ensure that qualified samples are used for transcriptome sequencing.

#### Library preparation for mRNA-Seq

A total of 1.5 µg RNA per sample was used as starting material for rRNA removal using the Ribo-Zero rRNA Removal Kit (Epicentre, Madison, WI, USA). Sequencing libraries were generated using the NEBNext R UltraTM Directional RNA Library Preparation Kit for Illumina R (NEB, USA) as recommended by the manufacturer and the index code was added to the attribute sequence of each sample. Briefly, fragmentation was performed at high temperature using divalent cations in NEBNext First Strand Synthesis Reaction Buffer (5X). First-strand cDNA was synthesized using random hexamer primers and reverse transcriptase. Second-strand cDNA synthesis was subsequently performed using DNA polymerase I and RNase H, and the remaining overhangs were converted to blunt ends by exonuclease/polymerase activity. After adenylation of the 3’ ends of the DNA fragments, NEBNext adapter proteins with hairpin loop structures were ligated to prepare for hybridization. To preferentially select inserts of 150–200 bp in length, library fragments were purified using AMPure XP Beads (Beckman Coulter, Beverly, USA). Then, 3 µl of USER enzyme (NEB, USA) was added to the size-selected, adaptor-ligated cDNA at 37 °C for 15 min prior to PCR. PCR was then performed using Phusion High-Fidelity DNA Polymerase, universal PCR primers, and Index (X) primers. Finally, PCR products were purified (AMPure XP system), and library quality was assessed on an Agilent Bioanalyzer 2100 and qPCR.

#### Library preparation for lncRNA-Seq

Using the same library preparation method as mRNA-Seq.

#### Clustering and sequencing

Index-encoded samples were clustered on the acBot Cluster Generation System using the TruSeq PE Cluster Kitv3-cBot-HS (Illumia) according to the manufacturer’s instructions. After cluster generation, library preparations were sequenced on the Illumina platform and paired-end reads were generated.

#### Data analysis

Clean RNA-seq reads were aligned to the sheep reference genome (Ovis_aries.Oar_v4.0.genome.fa) using HISAT2 v2.0.4 with default parameters. The mapped reads were subsequently assembled into transcripts and quantified as fragments per kilobase of transcript per million mapped fragments (FPKM) using StringTie v2.1.4. The resulting transcriptome assemblies were used for downstream identification and quantification of mRNAs and long non-coding RNAs (lncRNAs).Differential expression analysis between the high residual feed intake (H-RFI) and low residual feed intake (L-RFI) groups was performed using the DESeq2 R package (v1.10.1). Genes with an adjusted P value < 0.01 and an absolute log₂(fold change) > 1 were defined as differentially expressed genes (DEGs). Gene Ontology (GO) enrichment analysis of DEGs was carried out using the clusterProfiler R package (v4.4.4). The statistical enrichment of DEGs in Kyoto Encyclopedia of Genes and Genomes (KEGG) pathways was assessed using KOBAS software (Mao et al., 2005).Based on the gene expression profiles, weighted gene co-expression network analysis (WGCNA) was employed to construct co-expression networks and identify gene modules associated with the traits of interest. Key co-expression modules were further subjected to network visualization and topological analysis in Cytoscape v3.10.4.

## Results

### Differences in growth performance and feed efficiency of Dexin male lambs with different residual feed intake

As shown in Table 2, daily feed intake and RFI of Dexin male lambs in the H-RFI group were significantly higher than those in the L-RFI and M-RFI groups (*P* < 0.01), and the feed-to-weight ratio was also significantly greater in the H-RFI group compared to the other two groups (*P* < 0.05). No significant differences were observed among the groups for average daily gain or mid-term metabolic body weight (*P* > 0.05).

**Table 2:** Feed intake and growth performance characteristics of Dexin male lambs with different residual feed intake

Items	Group			SEM	*P* -value
	L-RFI	M-RFI	H-RFI		
The number of animals.	13	18	11		
DMI(kg/d)	1.61^Bb^	1.68^Bb^	1.83^Aa^	0.02	0.001
ADG(g/d)	0.314	0.316	0.331	0.01	0.73
Mid-term metabolic weight. (kg)	15.13	14.77	15.24	0.16	0.47
Initial weight. (kg)	31.16	29.96	31.18	0.50	0.52
Final weight. (kg)	43.73	42.62	44.41	0.62	0.51
FCR	5.03^b^	5.16^b^	5.54^a^	0.07	0.01
RFI	-0.13^Cc^	0.00^Bb^	0.11^Aa^	0.01	0.001

^ABC^Different superscripts indicate significant differences within a row (*P* < 0.01)

^abc^Different superscripts indicate significant differences within a row (*P* < 0.05)

### Differences in meat and wool quality of Dexin sheep with different residual feed intakes

As shown in Table 3, the cooked meat rate of fattening sheep in the L-RFI group was significantly higher than that of the H-RFI group (*P* < 0.05), while no significant differences were observed in other physical meat quality indicators (*P* > 0.05). According to Supplementary Table 1, cis-11-eicosenoic acid (C20:1) content in the left longissimus dorsi muscle was significantly increased in the L-RFI group (*P* < 0.05), whereas no significant differences were detected in other fatty acid indicators (*P* > 0.05). Supplementary Table 2 shows that the amino acid composition of the left longissimus dorsi muscle did not differ significantly between the two groups (*P* > 0.05).

**Table 3:** Differences in meat quality of Dexin fine-wool mutton with different residual feed intake

Item		Group		*P*-vaule
		H-RFI	L-RFI	
pH		6.72 ± 0.20	6.62 ± 0.37	*P* = 0.4
Cooked meat yield(%)		60.36 ± 0.01	64.38 ± 0.01	*P* = 0.04
Water loss(%)		18.89 ± 0.01	11.69 ± 0.03	*P* = 0.18
Water loss rate(%)		19.44 ± 0.03	25.36 ± 0.02	*P* = 0.18
Lamb color	a	9.00 ± 1.27	9.42 ± 1.92	*P* = 0.52
	b	6.65 ± 0.92	6.46 ± 1.13	*P* = 0.66
	L	32.86 ± 1.42	32.89 ± 2.10	*P* = 0.96

As shown in Table 4,Wool fiber diameter in L-RFI sheep was significantly reduced (*P* < 0.05), hair cluster length was significantly shorter (*P* < 0.01), and the straightening rate was significantly higher (*P* < 0.05), with no significant differences observed in other wool quality parameters (*P* > 0.05).

**Table 4:** Differences in wool quality of Dexin meat wool under different residual feed intake

Item		Group		*P*-vaule
		H-RFI	L-RFI	
Fiber diameter(um)		21.83 ± 1.37	20.06 ± 1.23	*P* = 0.045
A dense growth of fur(mm)		55.43 ± 4.24	45.80 ± 2.28	*P*<0.01
Number of crimps (per 2.5 cm)		10.29 ± 2.06	11.60 ± 1.67	*P* = 0.27
Elongation(%)		24.31 ± 2.70	30.12 ± 4.08	*P* = 0.01
Wool color depth	L	77.61 ± 1.68	78.25 ± 0.68	*P* = 0.44
	Wht	76.05 ± 1.47	76.51 ± 0.70	*P* = 0.53
	YI	19.58 ± 0.82	20.59 ± 1.08	*P* = 0.10

### Analysis of differential expressions of lnc-RNA and mRNA in the hypothalamus of Dexin sheep with different residual feed intakes

**Fig. 1:**
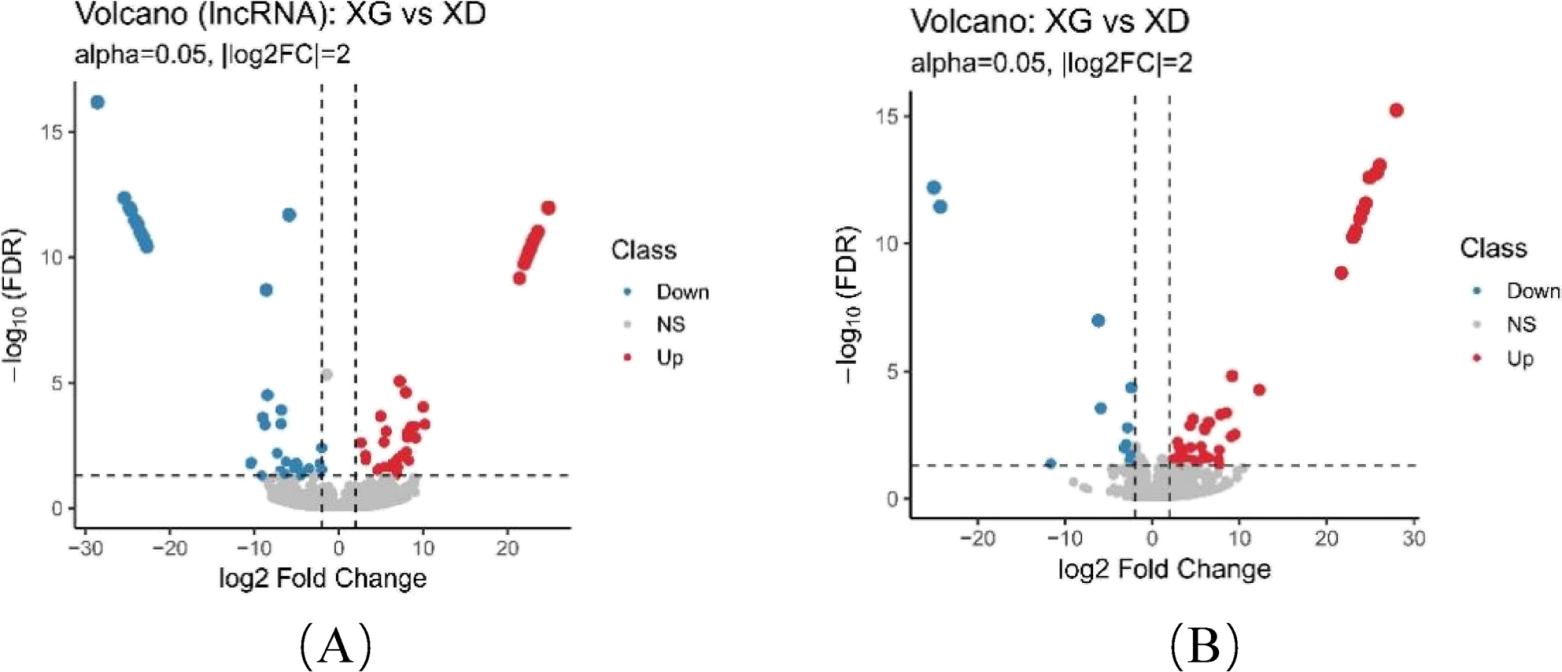
Volcano plot of differentially expressed genes in the hypothalamus of Dexin fine-wool sheep with different residual feed intakes: Lnc-RNA (**A**) and mRNA (**B**)

As shown in Fig. 1 (A), a total of 10,602 tested genes were screened, of which 100 genes were differentially expressed, 55 of which were upregulated and 45 of which were downregulated.As shown in Fig. 1 (B), a total of 24,500 tested genes were screened, of which 78 were differentially expressed, 66 were upregulated, and 12 were downregulated.

**Fig. 2:**
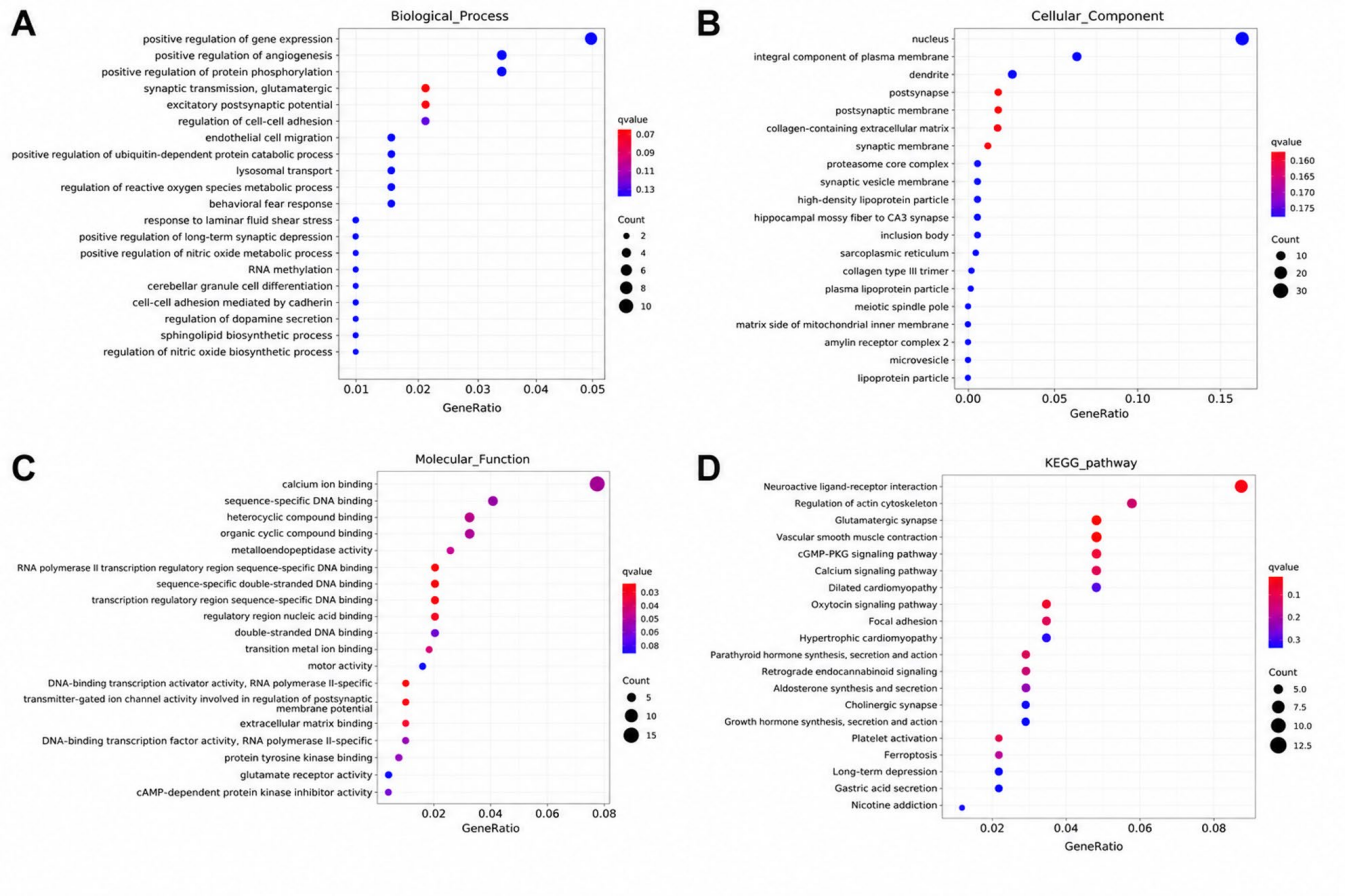
Analysis of hypothalamic mRNA in Dexin fine-wool meat sheep with different residual feed intake, (**A**) GO enrichment of Biological Process, (**B**) GO enrichment of Cellular Component, (**C**) GO enrichment of Molecular Function, and (**D**) KEGG pathway diagram

GO and KEGG enrichment analyses were performed on hypothalamic mRNAs to explore the functions of DEGs. In Biological Process, the H-RFI group was enriched in positive regulation of gene expression, angiogenesis, and protein phosphorylation (Fig. 2A). In Cellular Component, the top pathways included nucleus, plasma membrane, and dendrite (Fig. 2B). In Molecular Function, calcium ion binding, sequence-specific DNA binding, and heterocyclic compound binding were most enriched (Fig. 2C). KEGG analysis revealed enrichment in neuroactive ligand–receptor interaction, glutamatergic synapse, and calcium signaling pathways (Fig. 2D). These results indicate that H-RFI sheep may have higher feed intake than L-RFI sheep due to enhanced glutamatergic synaptic transmission, calcium signaling, and neurotransmitter receptor activity, along with inhibition of satiety-related pathways, collectively increasing neural excitability and appetite.

**Fig. 3:**
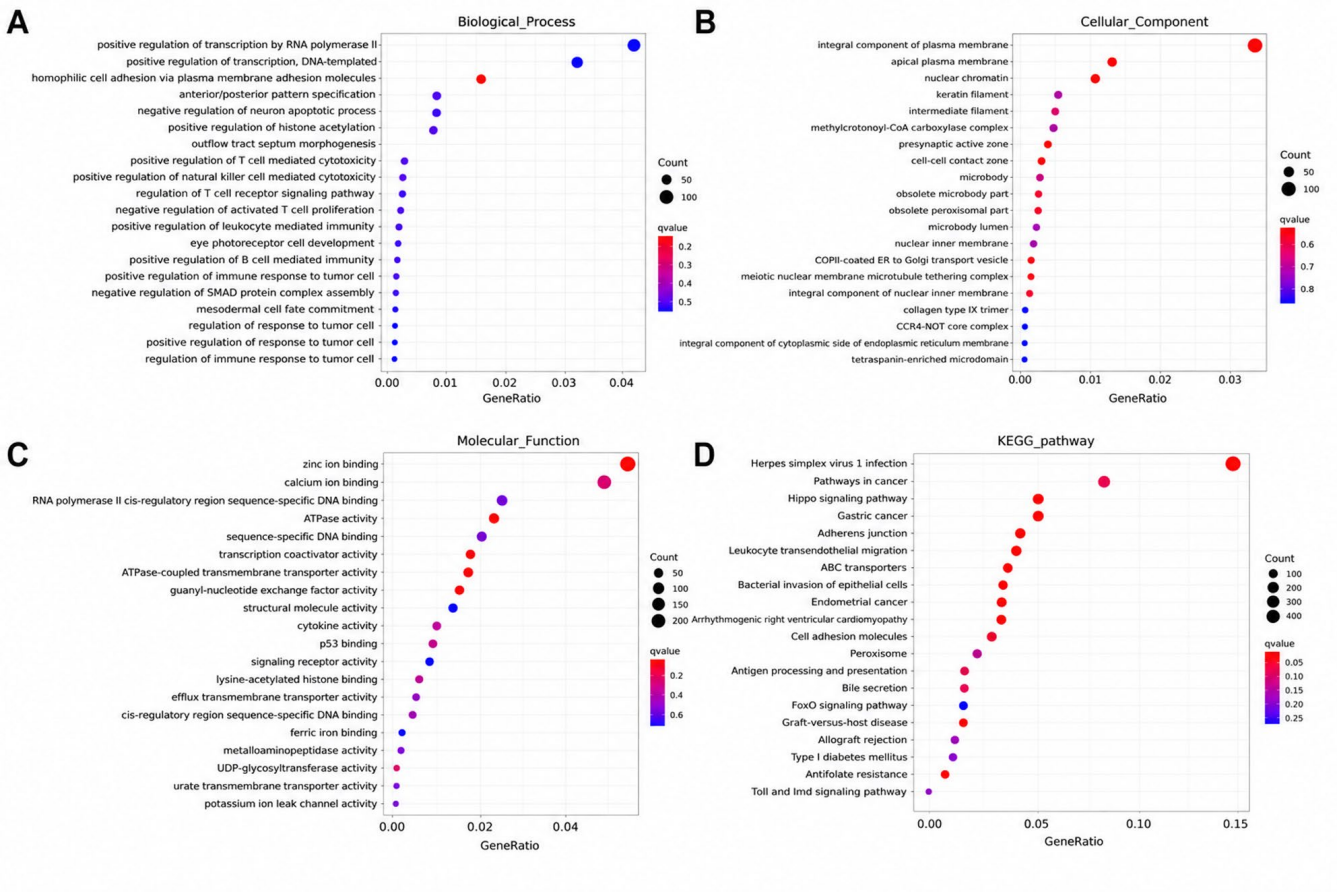
Lnc-RNA analysis of hypothalamus of Dexin fine-wool meat sheep with different residual feed intake, (**A**) GO enrichment of Biological Process, (**B**) GO enrichment of Cellular Component, (**C**) GO enrichment of Molecular Function, and (**D**) KEGG pathway diagram

Lnc-RNA analysis revealed that, in the H-RFI group, Biological Process enrichment included positive regulation of transcription by RNA polymerase II, DNA-templated transcription, and homophilic cell adhesion via plasma membrane adhesion molecules (Fig. 3A). In Cellular Component, the most enriched pathways were integral components of plasma membrane, apical plasma membrane, and nuclear chromatin (Fig. 3B). Molecular Function enrichment included zinc ion binding, calcium ion binding, and RNA polymerase II cis-regulatory region sequence-specific DNA binding (Fig. 3C). KEGG analysis indicated enrichment in herpes simplex virus 1 infection, pathways in cancer, and Hippo signaling pathway (Fig. 3D). These results suggest that high RFI sheep may exhibit a heightened immune response, which could consume excess energy and reduce growth performance.

### WGCNA analysis

**Fig. 4:**
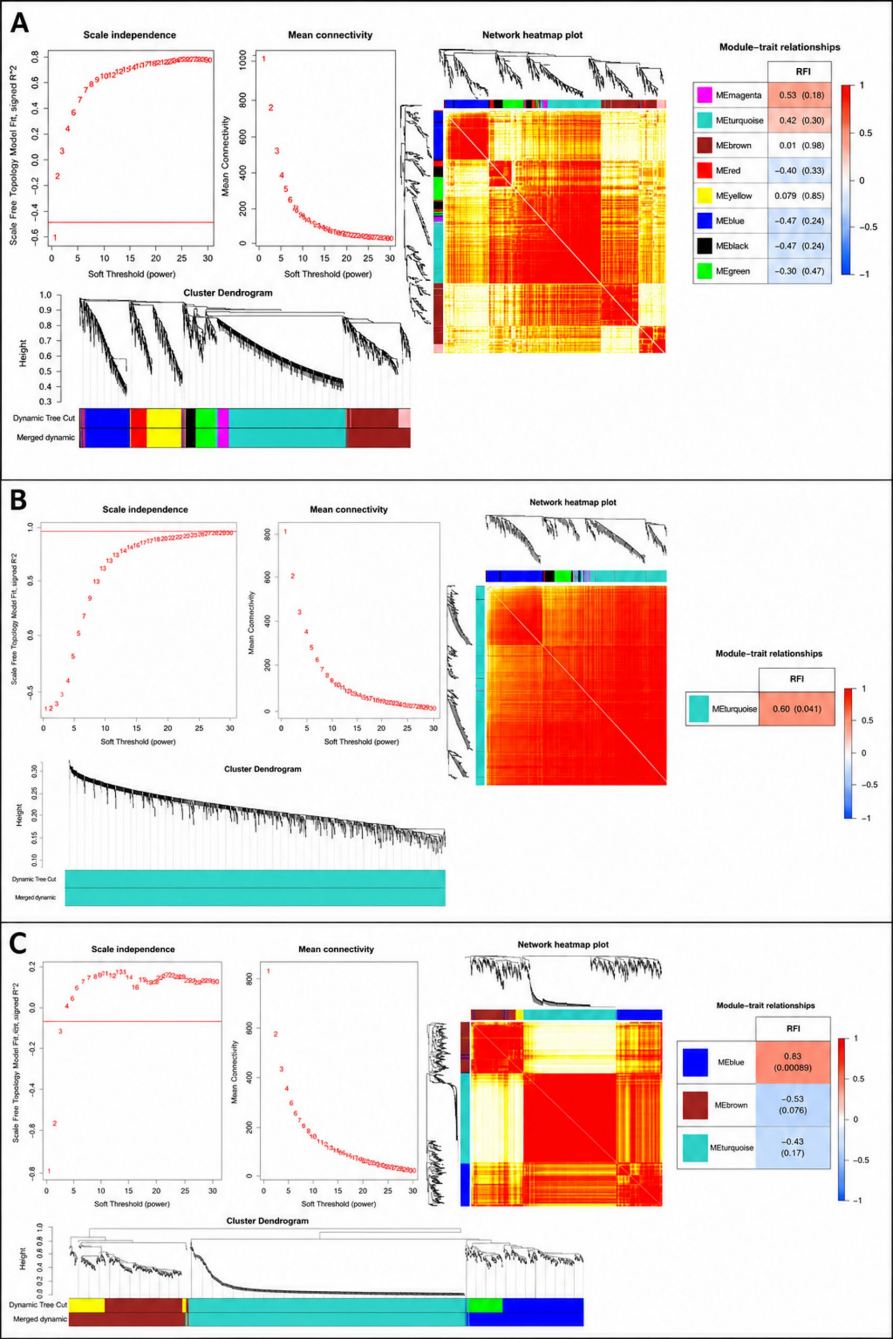
The top left figure illustrates network topology analysis under different soft-threshold powers. Left 1 shows the relationship between the scale-free fit index (y-axis) and soft-threshold power (x-axis). Left 2 shows the relationship between average connectivity (degree, y-axis) and soft-threshold power (x-axis). The bottom figure is a dendrogram of genes obtained by clustering differences based on consensus topology overlap, with the corresponding module colors indicated by color lines. Left 3 uses a heatmap to visualize the gene network. Lighter colors represent low overlap, and gradually deepening red represents high overlap. (**A**) Hypothalamus, (**B**) Rumen, (**C**) Duodenum

As shown in Fig. 4, with a correlation threshold of 0.99, WGCNA identified eight co-expression modules in the hypothalamus (A), where the “turquoise” module was the largest (618 genes), followed by the “brown” (308), “blue” (281), “yellow” (253), “green” (106), “red” (86), “black” (55), and “magenta” (25) modules; specifically, the magenta module showed the strongest positive correlation (*r* = 0.53) with RFI, while the black and blue modules exhibited the highest negative correlations (*r* = -0.47). In the rumen (B), a single “turquoise” module (537 genes) was positively correlated with RFI (*r* = 0.6), whereas in the duodenum (C), three modules were constructed, led by the “turquoise” (466 genes), “brown” (192), and “blue” (152) modules, with the blue module reaching the highest positive correlation (*r* = 0.83) and the turquoise module showing the most significant negative correlation (*r* = -0.53) with RFI.

### Analysis of mRNA in the duodenum of Dexin male lambs with different residual feed intake

**Fig. 5:**
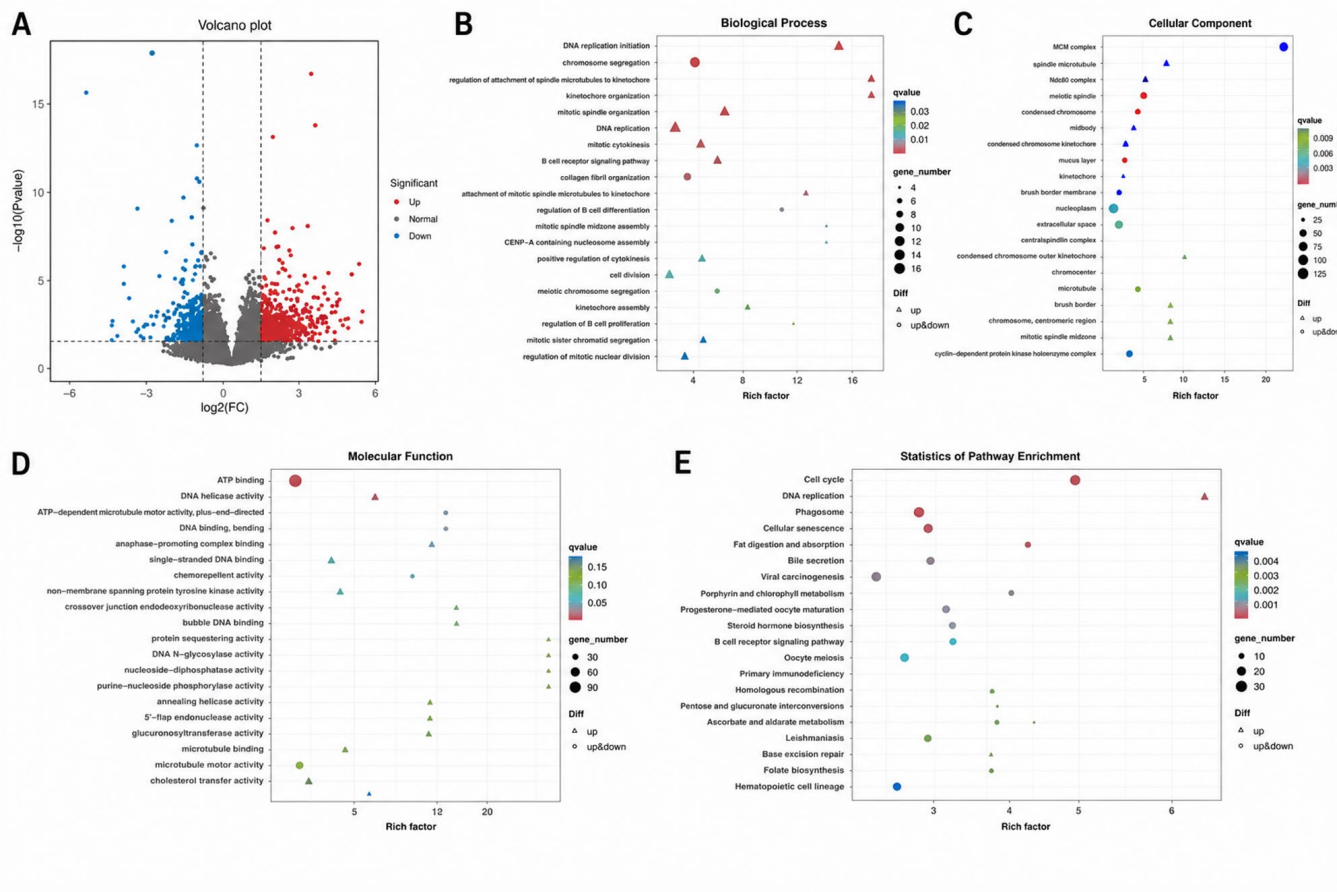
Analysis of duodenum mRNA in Dexin fine-wool meat sheep with different residual feed intake, (**A**) volcano map, (**B**) GO enrichment of Biological Process, (**C**) GO enrichment of Cellular Component, (**D**) GO enrichment of Molecular Function, and (**E**) KEGG pathway map

As shown in Fig. 5A, a total of 1,218 differentially expressed mRNAs were detected in the duodenum, including 802 upregulated and 416 downregulated genes. GO and KEGG enrichment analyses were performed to explore their functions. In the H-RFI group, Biological Process enrichment included DNA replication, cell cycle regulation, chromosome segregation, and spindle organization, with B cell immune response pathways also altered (Fig. 5B). In Cellular Component, the most enriched pathways were MCM complex, spindle microtubule, and Ndc80 complex (Fig. 5C). In Molecular Function, cholesterol transfer activity, microtubule motor activity, and DNA helicase activity were most enriched (Fig. 5D). KEGG analysis highlighted enrichment in cell cycle, DNA replication, and B cell receptor signaling pathways (Fig. 5E).

These results indicate that, compared with L-RFI sheep, H-RFI sheep exhibit activation of fat digestion and absorption pathways, enhanced cholesterol transport, and enrichment of brush border membrane structures, accompanied by active epithelial cell proliferation (cell cycle activation, MCM complex upregulation) and a distinct immune-metabolic regulatory network (B cell signaling and bile acid metabolism). This suggests that duodenal epithelial cells in H-RFI sheep may have accelerated metabolism and heightened immune response, similar to observations in the hypothalamus, implying that H-RFI sheep allocate more nutrients to cellular metabolism and immune function.

### Analysis of rumen mRNA in Dexin lambs with different residual feed intake

**Fig. 6:**
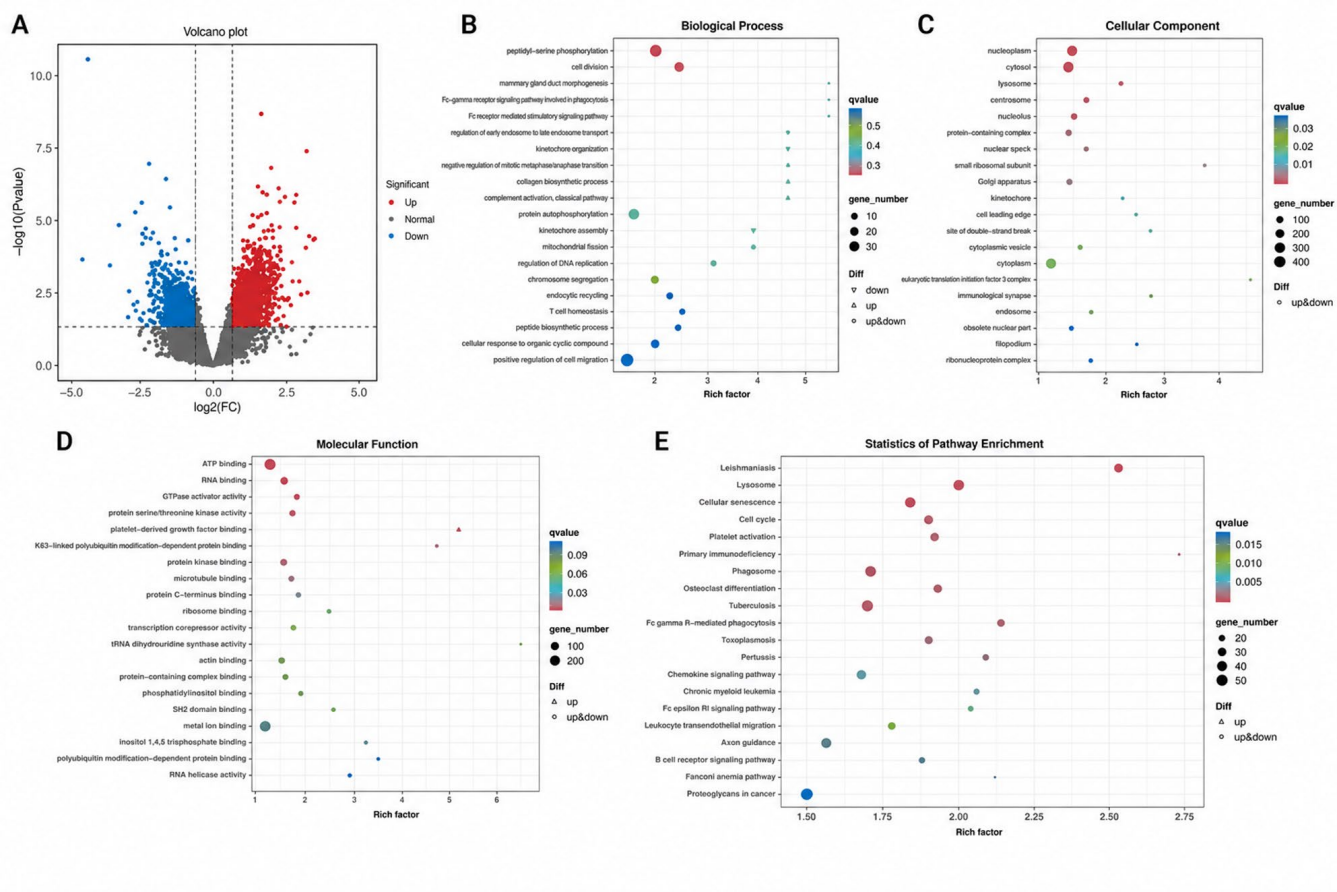
Rumen mRNA analysis of Dexin fine-wool meat sheep with different residual feed intake, (**A**) volcano plot, (**B**) GO enrichment of Biological Process, (**C**) GO enrichment of Cellular Component, (**D**) GO enrichment of Molecular Function, (**E**) KEGG pathway diagram

As shown in Fig. 6A, a total of 3,391 differentially expressed mRNAs were detected in the rumen, including 1,876 upregulated and 1,515 downregulated genes. GO and KEGG enrichment analyses were performed to explore their functions. In Biological Process, the main enriched pathways included peptidyl-serine phosphorylation, cell division, and mammary gland duct morphogenesis (Fig. 6B). In Cellular Component, enrichment was highest for nucleoplasm, protein-containing complexes, and sites of double-strand breaks (Fig. 6C). In Molecular Function, ATP binding, RNA binding, and GTPase activator activity were most enriched (Fig. 6D). KEGG analysis highlighted lysosome, cell cycle, and B cell receptor signaling pathways (Fig. 6E).These results indicate that, in H-RFI sheep, core rumen functions related to energy metabolism (ATP binding, lysosomal pathway) are significantly enhanced. Simultaneously, cell cycle pathways and nuclear and cytoplasmic components (e.g., MCM complex, kinetochore structures) are synergistically activated, promoting rapid renewal of epithelial cells and establishing a distinct immune regulatory pattern.

### Hypothalamic mRNA-LncRNA co-expression analysis

**Fig. 7:**
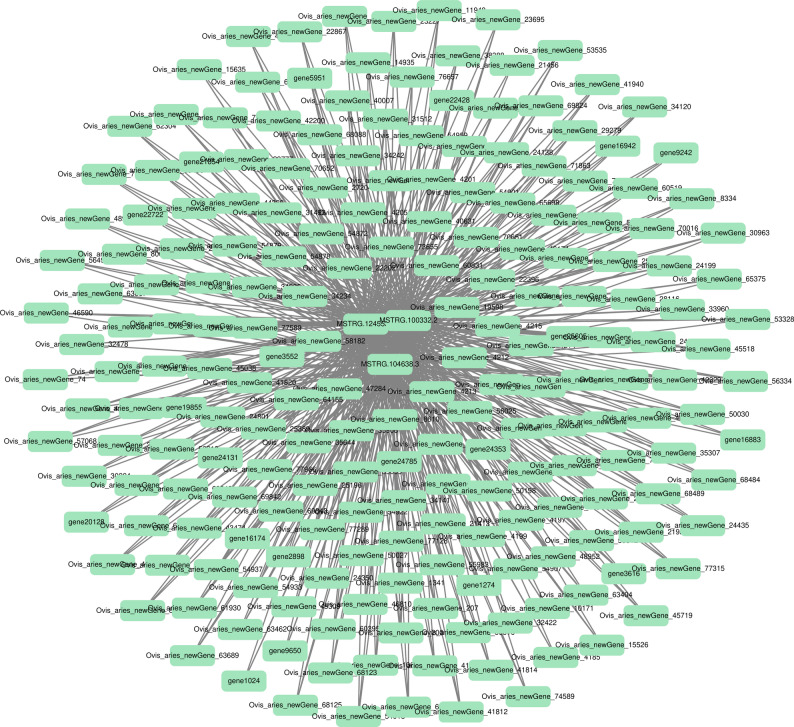
Hypothalamic LncRNA and mRNA co-expression network diagram

In the hypothalamus, based on transcriptome data from 10 samples (5 high-value and 5 low-value), the original counts matrices of mRNA and lncRNA were first compiled separately. Samples common to both types of transcripts were retained. The merged expression matrix was then normalized and subjected to variance stabilization transformation (VST) using DESeq2 to eliminate the influence of sequencing depth and differences between samples.Based on this, 100 significantly differentially expressed lncRNAs (DE-lncRNAs) were extracted from the differential analysis results, and Spearman correlation analysis was performed on their VST expression levels and the VST expression levels of all mRNAs.By calculating the correlation coefficient and p-value between each DE-lncRNA and all mRNAs, and performing multiple regression analysis (FDR) using the Benjamini–Hochberg method, with a correlation threshold of |r| ≥ 0.8 and FDR < 0.05, 4,785 significant lncRNA–mRNA co-expression relationships were obtained, involving 97 DE-lncRNAs and 1,265 mRNAs, thus constructing a hypothalamic lncRNA–mRNA co-expression network.Furthermore, the topological importance of each lncRNA in the network is measured by the number of mRNAs it is linked to (degree), the degree of nodes in the network is counted, and the DE-lncRNAs with the highest degree are selected as candidate “hub lncRNAs”.These hub lncRNAs and their co-expressed mRNAs were extracted into subnetworks and exported to Cytoscape for visualization (Fig. 7). Specific genes are listed in the Supplementary Table 2.

### Analysis of major functional pathways

**Fig. 8:**
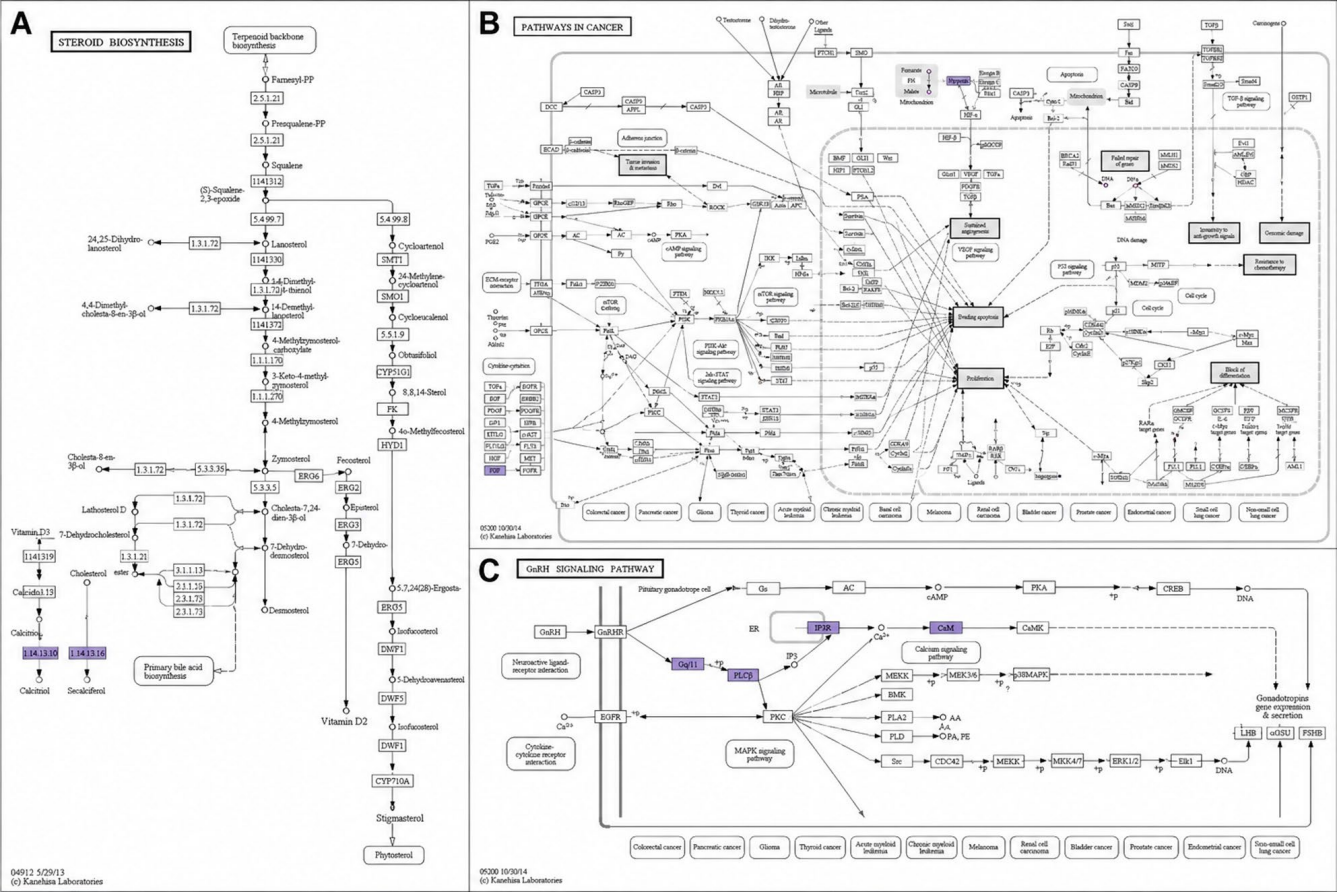
EGG pathway diagram of WGCNA co-expression analysis module, (**A**) hypothalamus, (**B**) duodenum, (**C**) rumen

As shown in Fig. 8A, WGCNA analysis of the hypothalamic transcriptome revealed that cholesterol 24-hydroxylase in the steroid biosynthesis pathway was upregulated in H-RFI sheep, potentially affecting the production of calciferol and agarol. In the duodenum, HIF prolyl hydroxylase in the HIF-1 signaling pathway was significantly upregulated in the H-RFI group (Fig. 8B), influencing the stability and activity of HIF-1α. Additionally, fibroblast growth factor expression in the cytokine–cytokine receptor interaction pathway was increased, suggesting enhanced cell proliferation, differentiation, migration, survival, tissue repair, and angiogenesis. In rumen tissue, multiple components of the GnRH signaling pathway, including MIVP2, MT1, Gq/11, PLCB, IP3R, and CaM, were upregulated in H-RFI sheep (Fig. 8C), affecting cell proliferation, differentiation, migration, and coordinating gonadotropin secretion.

## Discussion

According to previous research by our group, the levels of growth hormone, thyroid hormone and leptin in the serum of sheep in the L-RFI group were significantly lower than those in the H-RFI group, while the apparent digestibility of dry matter and crude protein was significantly higher [12]. The results suggest that individuals with low RFI exhibit specificity in energy and lipid metabolism and have enhanced feed digestibility. Regarding the gastrointestinal microbiota, the abundance of flora such as Anaerovoracaceae, Christensenellaceae_R_7_group, Proteobacteria, and Roseburia was significantly correlated with feed intake; Microbial function prediction further showed that the L-RFI group had upregulated expression in related pathways such as energy, amino acid, carbohydrate metabolism and cell migration [13]. These results suggest that sheep with different feed efficiencies may have a potential regulatory mechanism involving energy metabolism, protein turnover, and immune regulation, which ultimately affects their growth performance and livestock product quality.Based on the above findings, this study further predicted the mRNA function of the hypothalamus-gastrointestinal axis in sheep with different RFI and conducted meat and wool quality detection and analysis.

After excluding major influencing factors such as breed and sex, this study focuses on the genetic and physiological effects of residual feed intake (RFI) on the meat and wool quality of sheep.Meat physical properties, especially the yield of cooked meat, are key indicators for measuring water retention and juiciness; a higher yield of cooked meat usually means lower cooking loss and better taste quality [14, 15]. The results of this experiment showed that the cooked meat percentage of the German ram lambs in the low-RFI (L-RFI) group was significantly higher than that in the high-RFI (H-RFI) group, which is consistent with the conclusions of previous studies in fattening lambs and low-RFI Angus bulls [16–18]. Feed conversion efficiency is a key factor affecting wool yield. This study confirmed that the wool fiber diameter and tuft length of sheep with low residual feed intake (L-RFI) were significantly smaller than those of the high RFI (H-RFI) group.This finding is consistent with Carneiro et al. [19]’s report that L-RFI sheep have higher wool yield per unit area. Protein absorption efficiency is the core factor determining wool fiber development [20]. Our previous microbiome studies based on KEGG function have revealed that the gastrointestinal microbes of L-RFI sheep are more active in amino acid absorption and metabolism [21], which may be the key mechanism by which they improve wool quality by optimizing protein utilization.

The hypothalamus, as the energy regulation center of ruminants, not only senses the body’s energy state, but is also the core of regulating the balance between nutrient intake and energy metabolism [22–24]. This core role makes it an ideal target for exploring the dynamics of nutrition. LncRNAs, as non-coding RNA molecules, play a key role in epigenetic regulation, transcriptional control, metabolic and immune responses [25]. A recent study analyzed hypothalamic LncRNAs in Angus cattle, revealing that differentially expressed genes between individuals with high and low RFI are primarily involved in thermogenic and oxidative phosphorylation, improving efficiency by regulating mitochondrial function and energy utilization.This study identified 100 differentially expressed lncRNAs (55 upregulated, 45 downregulated) and 78 mRNAs (66 upregulated, 12 downregulated) through hypothalamic transcriptome analysis, suggesting potential synergistic regulation. GO enrichment analysis of hypothalamic lncRNAs in the high feed conversion ratio (H-RFI) group showed enrichment primarily in positive transcriptional regulation of RNA polymerase II, plasma membrane synthesis, and the calcium-zinc ion binding pathway.KEGG analysis revealed significant differences in RFI in calcium signaling pathways, postsynaptic membranes, and immune-related pathways.Among them, the results of calcium ion enrichment in H-RFI were similar to those of Jessica [26]. Current research shows that calcium ions have a significant synergistic effect with joint contraction and meat tenderness, which also indirectly proves why the longissimus dorsi muscle of sheep in the L-RFI group was less damaged by steaming.Plasma membrane synthesis is closely related to intracellular transport, including nutrient transport and signal recognition [27]. In this study, KEGG showed that the postsynaptic membrane pathway was enriched and upregulated, and neurotransmitters in the brain were released between synapses, which changed the excitability of the postsynaptic membrane and bound to receptors on the membrane, activating downstream signaling pathways and thus affecting the excitability of the central nervous system [28]. After activation of postsynaptic membrane receptors, the concentration of Ca2 + in the cytoplasm increases, thereby regulating ATPase activity [29]. This also coincides with the results of this experiment, where the calcium ion pathway was significantly upregulated in both Lnc-RNA and mRNA analysis results.mRNA results showed that the functional pathways were mainly enriched in gene positive regulation, plasma membrane integration, and neuroactive ligand-receptor interactions.The above results indicate that H-RFI sheep may upregulate the expression of receptors on the postsynaptic membrane and nerve cells through a series of differences in neurotransmitter secretion, and through Ca2 + ions, sheep with high residual feed intake exhibit stronger nerve excitability and appetite, leading to increased feed consumption.WGCNA and KEGG further revealed that the upregulation of cholesterol 24-hydroxylase in the steroid biosynthesis pathway of H-RFI sheep led to oxidative stress, consistent with previous findings that H-RFI sheep had lower antioxidant enzyme activity [13].

In the rumen (the main organ for feed fermentation and nutrient reception) [30, 31], 3391 differentially expressed genes were detected by mRNA (1876 upregulated and 1515 downregulated).GO enrichment analysis showed that peptidyl serine phosphorylation was significantly expressed in the H-RFI group, which is related to the regulation of glycolysis and post-mortem muscle metabolism [32]. WGCNA analysis revealed the upregulation of multiple genes in the GnRH signaling pathway, including MAPK interaction and spindle stabilizer protein 2. The main pathway affected by this pathway is the upregulation of Gq/G11 expression, which affects the upregulation of PLCβ expression, and the activation of IP3 receptor through secretion of IP3, which in turn increases the upregulation of CaM gene expression through CA2 + ions. This pathway suggests that H-RFI sheep may use more energy for the regulation of gonadotropins and reproductive processes [33], resulting in a decline in growth performance, which is similar to the study by Rebecca [34].

In the duodenum (the main site of amino acid and peptide absorption) [35], 3391 differentially expressed genes were identified (1876 upregulated and 1515 downregulated).GO and KEGG analyses showed that B cell-related pathways were enriched, including B cell immune response and B cell receptor signal transduction; Liu’s [36] study on the duodenal transcriptome of broilers with different residual feed intake showed that oxidative stress and inflammatory response were less in the duodenum of low RFI broilers, and their digestive and absorptive capacity was stronger, which is consistent with the results of this study.In ruminant studies, numerous experiments have shown that high and low residual feed intakes have differences in immune pathways, including differences in redox homeostasis and immune response pathways in liver transcriptomes of high and low feed intakes in Nellore bulls [37]; The transcriptome of Charolais bovine thoracolumbar longus muscle showed differential expression of genes related to immune function and inflammatory response [38]; In whole blood testing, different levels of remaining feed intake in Angus bulls showed enrichment differences in T cell regulatory signaling pathways and cell response to bacteria-related pathways [39]. The above results indirectly corroborate the results of the duodenum transcriptome analysis in this experiment, but no research has been found in the immune B cell pathway. This provides a new idea for the subsequent research on Dexin fine-wool sheep.KEGG highlights the upregulation of the HIF-1 signaling pathway, which is crucial for oxygen homeostasis [40]. Fibroblast growth factor in the cytokine-cytokine receptor interaction pathway further indicates enhanced cell proliferation, survival, and tissue repair [41]. These findings, combined with previous antioxidant activity assays [13], suggest that high feed intake (H-RFI) sheep exhibit higher immune activity and cellular metabolism, consuming more nutrients, which may limit their growth performance under the same feeding conditions, unlike low feed intake (L-RFI) sheep.

## Conclusion

This study shows that differences in residual food intake (RFI) are closely related to the neural signaling activity of the hypothalamus and the energy metabolism and cell renewal status of the gastrointestinal tract.High-RFI sheep exhibited higher levels of neural excitability, energy metabolism activity, and cell proliferation in the hypothalamus, rumen, and duodenum, suggesting that the intrinsic cost of maintaining basic physiological activities was higher, which may be a key mechanism leading to their lower feed efficiency. 

## Supplementary Information

Supplementary Material 1

Supplementary Material 2

## Data Availability

The data has been uploaded to the Zenodo database under 10.5281/zenodo.19500349.
